# Right Ventricular Ablation as a Therapeutic Option for Left Ventricular Hypertrabeculation / Noncompaction

**Published:** 2014-03-12

**Authors:** Josef Finsterer, Claudia Stollberger

**Affiliations:** 1 Krankenanstalt Rudolfstiftung; 2 2nd Medical Department, Krankenanstalt Rudolfstiftung, Vienna, Austria, Europe

**Keywords:** Right Ventricular Ablation, Left Ventricular Non-compaction Cardiomyopathy

With interest we read the article by Honarbakhsh et al. about the successful ablation of a right ventricular (RV) tachycardia in a patient with left ventricular hypertrabeculation, also known as noncompaction (LVHT) [1]. We have the following comments and concerns.

Though RV ablation is reported as having been successful, it would be interesting to know for how long the patient was followed-up after the procedure. This point is of particular importance since the patient is reported to have undergone RV ablation already before (ablation 1) the present procedure (ablation 2). How long was the interval between ablation 1 and ablation 2? For how long was ablation 1 successful?

A further point is the description of LVHT as a congenital condition. Though presumably correct for most of the cases, LVHT may not only be congenital, but also acquired, which means that LVHT either develops after birth (truly acquired) or is masked because of severe LV dilatation or thickening (hidden LVHT) [2,3]. When was LVHT actually detected in the presented patient? Was LVHT detected already before ablation 1? Were echocardiographies carried out before ablation 1?

A third point concerns the cause of LVHT. Nothing is mentioned if the patient carried any of the known mutations in genes so far reported in association with LVHT, such as TAZ, DTNA, ZASP, lamin A/C, MYH7, MYH7B, ACTC1, TNNT2, TNNI3, MYBPC3, TPM1, dystrophin, DMPK, ZNF9, LAMP2, GAA, mtDNA genes, AMPD1, GBE1, RYR1, COL7A1, PMP22, MMACHC, beta-globin, and DNAJC19 [Finsterer et al., submitted]. In this respect the authors should provide information if there was any indication of a neuromuscular disease or a chromosomal abnormality, conditions frequently associated with LVHT [4].

A fourth point is the familiarity of LVHT. Since LVHT is frequently associated with genetic defects, it is also frequently familial [5]. Were other family members investigated for LVHT? Was there any indication for hereditary disease in the patient's family? Was the family history positive for palpitations or were arrhythmias ever recorded in any of the first degree relatives of the patient?

The authors should also mention if there was previous stroke or embolism since the patient was 67 y at diagnosis of LVHT and assuming that LVHT was truly congenital it is quite likely that during such a long period a clinically manifesting or subclinical cerebrovascular embolic event had occurred. Did the patient ever undergo cerebral MRI to document old ischemic lesions in an embolic distribution?

Since cause and pathomechanism of LVHT are unknown, it is worthwhile to provide as much information as possible to clarify the many unsolved issues associated with LVHT. Though treatment of any complication of LVHT is symptomatic at the moment, deeper insight may give rise to the development of more causal therapeutic concepts.
